# Suggestions on Treatment of Puerperal Eclampsia and Post-Puerperal Kidney Lesions

**Published:** 1900-07

**Authors:** R. R. Kime

**Affiliations:** Atlanta, Ga., President Tri-State Medical Association Alabama, Georgia and Tennessee; ex-President Atlanta Society of Medicine.


### SUGGESTIONS ON TREATMENT OF PUERPERAL ECLAMPSIA AND POST-PUERPERAL KIDNEY LESIONS.*

By R. R. KIME, M.D., ATLANTA, GA.,

President Tri-State Medical Association Alabama, Georgia and Tennessee; ex-President Atlanta Society of Medicine.

The etiology and pathology of puerperal eclampsia is an unsettled question at present. A few years since it was generally considered due to uremia; now most writers agree that it is a toxemia.

As to how and where the toxic material is developed there is difference of opinion and various theories.

From clinical experience we learn that death of the fetus materially lessens the danger of eclampsia, which is certainly strong presumptive evidence that the living fetus elaborates a toxic material which is a factor in the production of eclamptic seizures.

We also learn that emptying the uterus in most cases helps to relieve the patient.

As soon after labor as nature can adjust herself to the changed conditions, a process of elimination is instituted and a return to the normal established, provided none of the vital organs are so seriously impaired that they cannot perform their functions.

It is claimed by some of late that the physiological hyperplasia of the thyroid gland during pregnancy acts as a kind of safety valve in the destruction of the toxins developed by the uterine metabolism during pregnancy, thereby preventing eclampsia and serious impairment of the kidneys and other organs.

If this be true, clinical experience in the future will demonstrate the utility of administering thyroid extract to these cases, at least as a prophylactic measure.

---

* Read at Georgia State Medical Association, April, 1900, Atlanta.

The presence of albumen in the urine during pregnancy does not always indicate serious trouble; neither does its absence preclude the possibility of eclampsia.

I have had three cases within the last eighteen months in my own practice in which there was no previous evidence symptomatically or in the urine to indicate impending danger. Two were primiparæ, one thirty-eight years, the other twenty-one years of age; both had convulsions during labor; a third, multipara, delivered of twins, and eight hours after delivery convulsions developed. After convulsions, in each case albumen appeared in urine and later sugar. All three recovered.

In controlling convulsions veratrum viride, Norwood's tincture, stands at the head as being safest and most certain. It not only controls convulsions, but favors elimination by diuresis and diaphoresis. It depresses the motor centers in the spinal cord, lessens the pulse rate, reduces temperature, conserves the vital forces of the patient and lessens the tendency to infection; while remedies that depress or interfere with elimination favor septic invasion. Veratrum should be used hypodermically at first in five- to ten-drop doses, later combined with a diuretic such as elixir, buchu, juniper and acetate of potash, given at regular intervals per os to prevent return of convulsions and increase elimination.

Chloroform should be used for temporary control of convulsions. Apomorphia is a prompt, reliable remedy, but it depresses and nauseates patient.

Venesection depresses, weakens vitality, favors infection, and should rarely be used.

Morphia checks elimination, increases stupor, and is uncertain. Chloral and bromides are uncertain but good adjuvants.

Veratrum administered as indicated above and repeated in sufficient doses to hold pulse near the normal range, with rapid clearing out of alimentary canal by use of calomel and salines, establishing elimination by kidneys, skin, liver and alimentary canal, puts patient in most favorable condition for recovery. Emptying the uterus is an important consideration in these cases.

Authorities class the death-rate at 25 to 35 per cent. previous to delivery, and at 7 per cent. in *post-partum* eclampsia. So it must be considered that the danger of death is practically over in a great many cases when the patient is delivered.

Yet judgment and discrimination must be exercised and not add too much to the exciting cause of convulsions nor lessen the patient's chances of recovery by a too rapid or heroic method of delivery.

Hysteria, epilepsy and apoplexy must be excluded; then good practice would be, in all cases where cervix is not sufficiently dilated for safe forceps delivery, nor comparatively easily dilated by fingers and hand, to wait until parts are properly prepared for labor. In such cases cleanse and disinfect vagina and cervix, carry a strip of iodoform gauze aseptic or made antiseptic up into uterine cavity with uterine dressing forceps as far as it will go, pack lower uterine segment and cervix snugly with the continued strip of gauze. Then dust boric acid into vagina freely and pack same with gauze and absorbent cotton. All gauze used should be dusted with boric acid, and if too dry to use easily, moisten with a weak carbolized solution.

This will institute labor, soften cervix, establish drainage from uterus, and render it possible to complete labor safely in twelve to twenty-four hours in most cases.

While waiting control convulsions as above indicated, clear alimentary canal, give diuretics, establish elimination by all means possible at command. The incision of cervix and mutilating operations are rarely, if ever, justifiable.

The prophylaxis and indications for emptying uterus in threatened eclampsia do not come within the scope of this paper.

The etiology and pathology are briefly mentioned only where they bear direct relation to treatment.

The ordeal of eclampsia and labor having passed, most cases are fairly convalescent by the time the lying-in period is passed. Yet in a goodly number serious lesion of the kidneys remain, and no obstetrician has discharged his full duty to his patient that does not watch and treat these cases until not only all albumen but all blood, pus, kidney epithelium and casts disappear from the urine.

I am fully convinced that in many instances where kidney lesions develop during pregnancy, in cases with and without eclampsia, that proper care and treatment will prevent permanent lesions.

After a trial of various remedies in these caaes, I have adopted the following general outline of treatment:

Peroxide of hydrogen is given in one-half to teaspoonful doses n as much hot lithia water as patient can drink, taken a short time before meals.

A diuretic tablet containing one grain each of pulverized digi-alis, squills, nitrate potash and buchu is given three times a day, with plenty of lithia water to flush out kidneys.

A bitter tonic is given at meal time. The following I find most beneficial:

| | |
|---|---|
| Strych. nit | gr. s.s. |
| Fluid hydrastis colored (Merrill's), | |
| Glycerin | aa ℥ ss. |
| Listerin | ℥ iii. |
| Lactopeptin syr. with phosphates, q. s | ℥ viii. |

M. Sig.: Two teaspoonfuls in a little water at eating.

Bowels are kept in a laxative condition by remedies best suited to the individual case.

In weak anemic cases some form of iron should be given.

The peroxide of hydrogen I consider one of the essentials in treating these cases. It clears out and cleanses the stomach, and has a special effect in clearing up the pus, casts and blood in the urine. Some authorities claim it is eliminated by the kidneys. If that be true, then its action on pus coming from kidneys in these cases, or bladder in case of cystitis, is explained.

It would necessarily be diluted by the time it reaches the kidney or bladder; hence slow in disintegrating pus corpuscles, and the evolution of gas so limited as not to produce deleterious effects. When not too strong has but little effect on vital tissues.

The action of the other remedies needs no comment except to emphasize the action of the fluid hydrastis. It is generally recognized that impaired digestion, overeating, especially of heavy diet or such food as tends to produce lithemia, predispose to or aggravate kidney lesions.

A restricted diet, with improvement of digestion and assimilation secured by use of hydrogen dioxide and bitter tonic, are potent factors in the relief of these cases, Systematic lung expansion and daily baths properly adjusted to each individual case supply oxygen to blood, tone up nervous system, favor oxidation in the tissues, increase cutaneous elimination, all of which lessen irritation and work of kidneys.

In conclusion I briefly report three cases:

Case 1.—Called in consultation to see Mrs. S., aged 30. Multipara, full term, having convulsions, in second stage of labor; gave ten drops veratrum, Norwood's tincture, hypodermically; delivered with forceps; child dead and decomposing; carried hand up into uterus to remove bits of decomposed membranes, etc.; irrigated, disinfected uterine cavity, applied antiseptic vulval dressing with binder. After my arrival patient had two convulsions before and two after delivery, one occurring as I entered room.

Veratrum was repeated and patient kept under its influence for some days.

Urine coagulated almost solid, contained pus, large hyaline and some granular casts, deficient in urea, very pale, low specific gravity. Patient had all the characteristic symptoms of profound toxemia. After months of treatment the eye symptoms of the kidney lesion cleared up. She became pregnant eighteen months afterward, was carried through full-term labor and delivered of dead child without special complication or return of kidney lesion. At no time did albumen appear, no casts, no toxemia discoverable.

Case 2.—In consultation. Mrs. R., aged 28 years. Multipara, eight months pregnant, in convulsions, semi-conscious; convulsions controlled with veratrum; cathartics and diuretics given.

Patient delivered next morning; urine coagulated almost solid with heat and acid; specific gravity 1008; large hyaline casts, pus, blood, kidney epithelium; symptoms of profound toxemia; almost complete loss of vision; eyes examined by specialist, confirming albuminuric retinitis. Under ordinary treatment for some weeks failed to improve, then placed on pyrozone, diuretic and bitter tonic; the urine soon cleared up, pus, albumen and casts disappearing. Vision gradually returned to near former standard. This patient also became pregnant about one year afterward and was delivered at full term without special complication.

Case 3.—Mrs. P., aged 28 years. Primipara; saw her first three months after delivery, giving history of kidney trouble with profound toxemia at labor and afterward; now weak, anemic, pulse 120 to 130; heart murmur of anemia, dyspnea marked, propped up in bed to get any relief; temperature sub-normal; edema of extremities; abdomen distended, approximately two gallons of fluid in peritoneal cavity; urine pale, specific gravity 1005, deficient in quantity, and urea, albumen, pus, and a few granular casts present.

Under treatment urine cleared up, fluid in peritoneal cavity absorbed and eliminated, heart murmur ultimately disappearing. After fluid was absorbed, local treatment corrected a subinvoluted, retroverted, slightly adherent uterus.

Patient became stout and hearty. Sixteen months afterwards became pregnant; in about six weeks took typhoid fever; while convalescing, nausea and vomiting of pregnancy set in and proved very, very obstinate. At no time was there any evidence of kidney lesion or heart complication except a general weakness. The president of this society saw the patient, and advised emptying uterus. After waiting a few days longer with a hope of getting relief otherwise, I rapidly dilated cervix, emptied, disinfected and tamponed uterus in twenty minutes, with very little loss of blood. Work done without anesthetic.

Patient gradually improved and made uneventful recovery.

